# Unusual case of chronic recurrent multifocal osteomyelitis

**DOI:** 10.1186/s12969-018-0267-4

**Published:** 2018-07-27

**Authors:** Ausra Snipaitiene, Rima Sileikiene, Justina Klimaite, Edita Jasinskiene, Rimantas Uktveris, Lina Jankauskaite

**Affiliations:** 1 The Lithuanian University of Health Sciences, A. Mickevičiaus g. 9, Kaunas, Lithuania; 2 The Hospital of Lithuanian University of Health Sciences Kauno Klinikos, Eiveniu g. 2, Kaunas, Lithuania

**Keywords:** CRMO, C-ANCA, Renal vasculitis, GPA, Hyperparathyroidism, Paediatric, Auto-inflammation, Autoimmune

## Abstract

**Background:**

Chronic recurrent multifocal osteomyelitis (CRMO) is a rare auto-inflammatory bone disorder that primarily affects young girls, with a mean age of 10 years at onset. Generally, it is a self-limited disease. However, recent data indicate that more than 50% of patients have a chronic persistent disease and about 20% a recurring course of this condition. Also, there are more cases reported with associated auto-inflammatory and autoimmune diseases. In this case report, we present a rare case of sporadic CRMO in which the patient eventually developed C-ANCA (cytoplasmic anti-neutrophil cytoplasmic antibodies)-associated renal vasculitis and hyperparathyroidism.

**Case presentation:**

A 14 year old female patient was brought to the emergency department with a sudden onset of left leg pain and oedema. After physical evaluation and initial investigation, she was diagnosed with femoral and pelvic deep vein thrombosis. While searching for possible thrombosis causes, osteomyelitis of the left leg was identified. Additional CT and MRI scans hinted at the CRMO diagnosis. Due to the multifocal lesions of CRMO, endocrinological evaluation of calcium metabolism was done. The results showed signs of hyperparathyroidism with severe hypocalcaemia. Moreover, when kidney damage occurred and progressed, a kidney biopsy was performed, revealing a C-ANCA associated renal vasculitis. Treatment was started with cyclophosphamide and prednisolone according to the renal vasculitis management protocol. Severe metabolic disturbances and hyperparathyroidism were treated with alfacalcidol, calcium and magnesium supplements. Secondary glomerulonephritis (GN) associated hypertension was treated with ACE (angiotenzine converting enzyme) inhibitors. Anticoagulants were prescribed for deep vein thrombosis. After 1.5 years of treatment, the patient is free of complaints. All microelement and parathormone levels are within normal range. Kidney function is now normal. To date, there are no clinical or diagnostic signs of deep vein thrombosis.

**Conclusions:**

This case report presents a complex immunodysregulatory disorder with both auto-inflammatory and autoimmune processes. We hypothesize that the long lasting active inflammation of CRMO may induce an autoimmune response and result in concomitant diseases like C-ANCA-associated vasculitis in our patient. Any potential specific pathogenic relationships between these two rare pathologies may need to be further studied. Furthermore, there is a lack of specific biomarkers for CRMO and more studies are necessary to identify CRMO’s characteristic patterns and how to best monitor disease progression.

## Background

Chronic recurrent multifocal osteomyelitis (CRMO) is a rare auto-inflammatory bone disorder that primarily affects young girls, with a mean age of 10 years at onset [1, 2]. The disease can cause multiple bone lesions, mostly affecting long bones of the lower extremities and vertebrae [3]. Generally, it is a self-limited disease; however, recent data show that more than 50% of patients have a chronic persistent and about 20% have a recurring course of this condition [1]. According to the Eurofever registry [4], there are only slightly more then 460 cases registered world-wide so far, with a prevalence of 1–9/million. [5]. As CRMO is the diagnosis of exclusion and can imitate other inflammatory bone conditions, it is thought to be underestimated [6]. Also, there are more cases emerging associated with other auto-inflammatory and autoimmune diseases, for example related to disorders with skin diseases [7, 8], peripheral arthritis [9–11], concomitant inflammatory bowel disease [12, 13], granulomatosis with polyangiitis (GPA, formerly known as Wegener granulomatosis) [14, 15] and others [16–19]. An association with several auto-inflammatory conditions can be detected in about one-third of CRMO patients [3]. Despite being well-known for about 50 years, CRMO still does not have approved treatment guidelines as the aetiology is unknown, and none of the therapies used currently have provided consistent outcomes. In this case report, we present a rare case of prolonged sporadic CRMO which eventually developed cytoplasmic anti-neutrophil cytoplasmic antibody (C-ANCA)-associated renal vasculitis and hyperparathyroidism.

## Case presentation

A 14-year-old female patient presented to the emergency department with a sudden onset of left leg pain and oedema. She had a fever of 38 °C once a few days before. Physical evaluation revealed a swollen and painful left leg with reduced range of motion of the left hip and bumps palpated on the right tibia proximal metaphysis. No signs of arthritis, possible intestinal inflammation or skin changes were seen during physical evaluation. The patient’s blood pressure and urinary output were normal. After the emergency ultrasound exam revealed a diagnosis of femoral and pelvic deep vein thrombosis, she was admitted to the hospital. According to her parents, the patient had had no chronic diseases to date. They indicated that she had a foot fracture at the age of 8 years. There were no autoimmune or auto-inflammatory disorders in the patient’s family history.

**Fig. 1:**
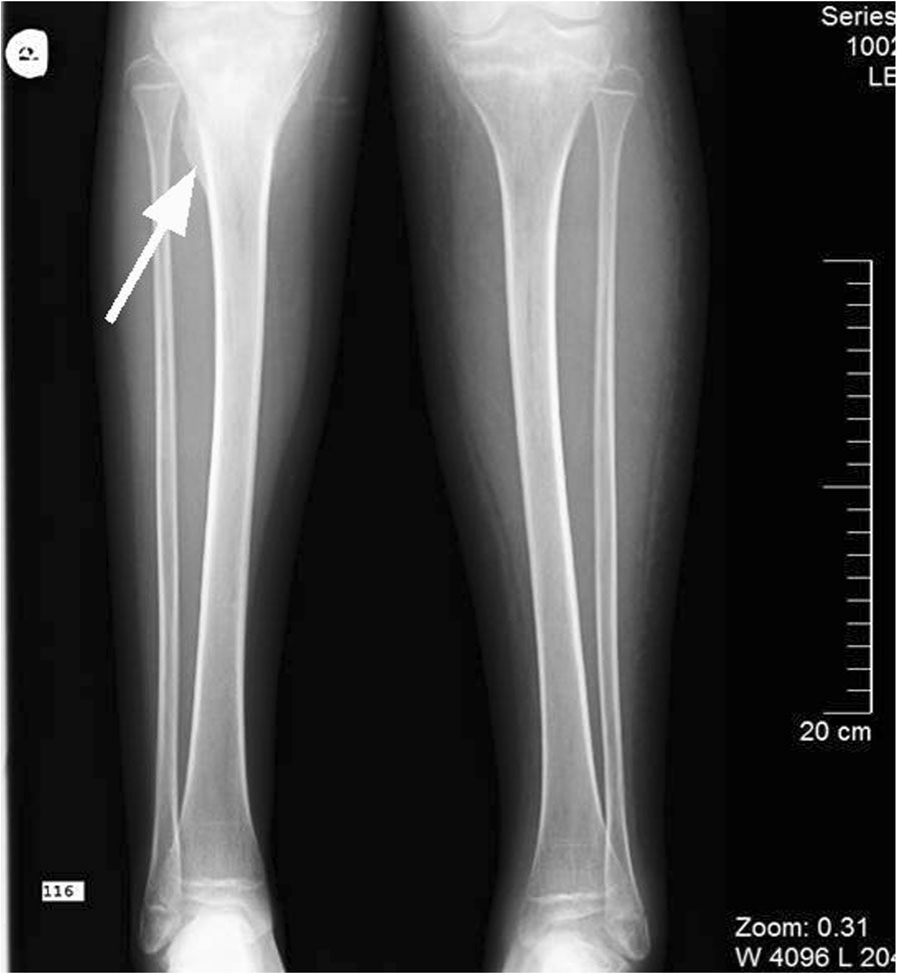
X-ray of the legs.Hyperostosis on the left tibia metaphysis is indicated by a white arrow

Initial investigations revealed high levels of inflammatory markers (CRP and ESR), and a severe microcytic anaemia as well as thrombocytosis, hypoalbuminemia and elevated fibrinogen concentration and D-dimer values (Table 1). Suspecting an infectious cause, a urinalysis was performed, and haematuria and proteinuria were detected. Both blood and urine cultures were sterile. X-rays of the legs were done (Fig. 1), showing sites of hyperostosis and sclerosis in the metaphysis of the right tibia along with a periosteal reaction, suggesting a possible osteomyelitis or oncological processes in the bones. As the diagnosis of acute osteomyelitis could not be disproved, broad-spectrum antibiotics were prescribed. However, the CRP level did not change significantly over the first few days, so other causes of acute thrombosis and inflammation were investigated. An abdominal ultrasound showed a giant, homogenous pelvic mass and hydronephrosis of the left kidney. Kidney function was quite abnormal as the creatinine level was 185 μmol/L and urinary protein excretion of 7 g over 24 h was detected. Within the next few days the patient’s kidney function deteriorated further, with a creatinine value increasing to 307 μmol/L with poor urine output and high blood pressure. The patient had no prior history of kidney problems.

**Table 1:** Blood tests results

Test	Result	Reference value
CRP (mg/L)	249.19	0–7.5
ESR (mm/h)	125	0–11
Complete blood count:		
WBC (X10^9^/L)	15.44	4.2–9.4
Neutrophil count (X10^9^/L)	12.42	1.8–7.5
Hb (g/L)	45	108–133
Erythrocyte (X10^12^/L)	2.81	3.9–4.9
MCV (fL)	62.6	77–91
MCH (pg)	17.1	24.8–30.2
Platelet count (X10^9^/L)	676	194–345
Albumin (g/L)	21	31–48
Fibrinogen (g/L)	7.63	2–4
D-dimers (mg/L)	7.87	0–0.5
Creatinine (μmol/L)	185 → 307	26.5–88.4
eGFR (ml/min/1,72m^2^)	33.5 → 20.2	90–120
BUN (mmol/L)	12.3	2.9–7.1
Feritine (μg/L)	448	15–150

*CRP* C-reactive protein, *ESR* erythrocyte sedimentation rate, *WBC* white blood cells *Hb* hemoglobin *MCV* mean corpuscular volume, *MCH* mean corpuscular hemoglobin, *eGFR* estimated glomerular filtration rate, *BUN* blood urea nitrogen

**Fig. 2:**
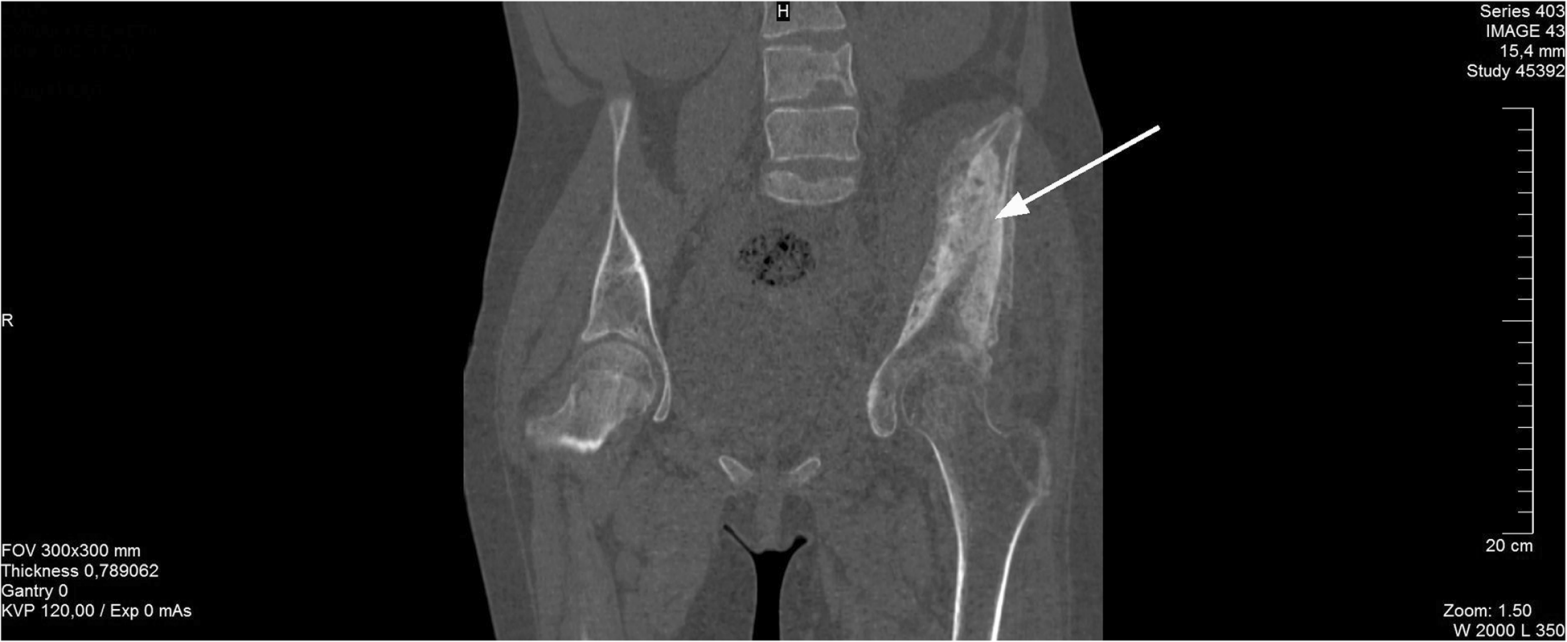
Whole-body CT (pelvis region) showing destruction and sclerosis of the left hip bones

Due to the findings of the abdominal ultrasound and leg X-ray, the child underwent a whole-body computed tomography (CT) scan. Multiple bone lesions were observed, the most severe of which were located in the left scapula, the fifth rib projection near the spine and a large deformity of the left pelvic bone close to the acetabulum (Fig. 2). The left iliopsoas muscle also appeared to be abnormal. Moreover, renal parenchymal thickening and oedema were found. Investigations for possible endocrine disorders were performed (Table 2). Signs of hyperparathyroidism were present together with hypocalcaemia, hypomagnesaemia and a low vitamin D3 concentration, as well as hypocalciuria in the 24-h urine test and hyperphosphaturia based on phosphate fractional excretion (Table 3).

**Table 2:** Endocrine function tests results

Test	Result	Reference value
Ca (mmol/L)	1.9	2.23–2.58
Ionized Ca (mmol/L)	0.83	1.28–1.48
P (mmol/L)	1.34	1.07–2
Mg (mmol/L)	0.72	0,74-1,03
Vitamin D3 (25-OH) (nmol/L)	27.6	< 50 – deficiency, 51–69 – insufficiency, 70–250 – normal, > 250 – intoxication
PTH (pmol/L)	32.69	< 6.74
TSH (pmol/L)	2.6	0.4–3.6
FT4 (pmol/L)	19.03	10–19
FT3 (pmol/L)	3.39	3.34–5.14
Anti-TPO (kU/L)	< 3	0–3.2

*Ca* calcium, *P* phosphorus, *Mg* magnesium, *PTH* parathyroid hormone, *TSH* thyroid-stimulating hormone, *FT4* free thyroxine, *FT3* free triiodothyronine *Anti-TPO* anti-thyroid autoantibodies

**Table 3:** Urine test results

Test	Result	Reference value
Erythrocytes (/μL)	12,166	< 10
Leukocytes (/μL)	433	< 10
Protein (g/L)	3	< 0,1
Protein/24 h (g)	7	< 1
Creatinine (μmol/L)	3884	
Ca (mmol/24 h)	< 0.37	2.5–7.5
Fractional Ex of Ca (%)	1.54	1–2
P (mmol/24 h)	11.47	12.9–42
Fractional Ex of P (%)	38.59	10–20

*Ca* calcium, *Ex* excreation *P* phosphorus

**Fig. 3:**
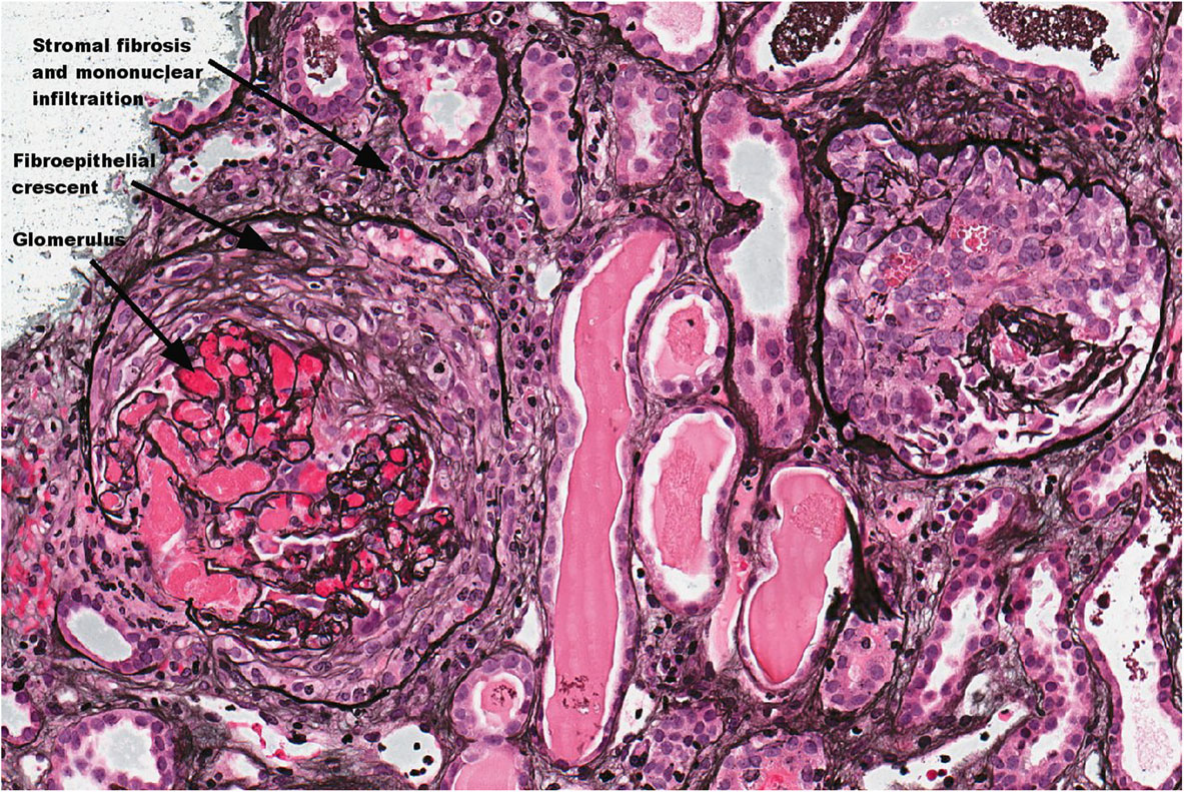
Histological image of kidney tissue. PAS-stained sample, 400× magnification. The crescentic glomeruli, stromal fibrosis, normal mesangial region are indicated by arrows

Bone and left kidney biopsies were performed. The bone biopsy from the affected site of the left pelvic bone demonstrated intertrabecular stromal fibrosis, several epithelioid granulomas with a central zone of necrosis and polymorphonuclear cells. Moreover, histological evaluation showed a few sites with plasma cell infiltration, including some cells positive for immunoglobulin G (IgG) and immunoglobulin G4 (IgG4). Histological examination of the surrounding connective tissue found mucoid oedema. The renal biopsy revealed an acute and active crescentic glomerulonephritis (GN) with ANCA-associated vasculitis (Fig. 3). Special staining was performed for the kidney sample, and no IgG subclasses were found.

Due to the results of left kidney biopsy together with thrombosis of the deep veins, additional screening for autoimmune diseases was carried out. The child tested positive for antinuclear antibodies (ANA 1:100), antibodies against centromere protein B (anti-CENP B), antibodies against proliferating cell nuclear antigens (anti-PCNA) and C-ANCA (Table 4). High levels of ferritin were also found (448 μg/L), indicating the possibility of both autoimmune and thrombotic causes of anaemia. However, antiphospholipid antibodies were not detected. Concerning the bone biopsy results, serum IgG levels were tested and only IgG4 was slightly increased (Table 4).

**Table 4:** Autoimmune markers

Marker	Result	Reference value
ANA	1+ (1:100)	Negative < 1:40
Anti-CENP B	1+	Negative
Anti-PCNA	1+	Negative
C-ANCA	1+ (1:10)	Negative
P-ANCA	Negative	Negative
Anti-dsDNA (kU/L)	3.13	< 12
Complement C3 (g/L)	1.42	0.79–1.52
Complement C4 (g/L)	0.26	0.16–0.38
IgG (g/L)	24.09	7.9–16.4
IgG4 (g/L)	2.98	0.035–2.3

*ANA* antinuclear antibodies, *Anti-CENP B* antibodies against centromere protein B, *Anti-PCNA* antibodies against proliferating cell nuclear antigens, *C-ANCA* cytoplasmic anti-neutrophil cytoplasmic antibodies, *P-ANCA* perinuclear anti-neutrophil cytoplasmic antibodies, *Anti-dsDNA* anti-double stranded DNA antibodies, *IgG* immunoglobuline G, *IgG4* immunoglobuline G4

**Fig. 4:**
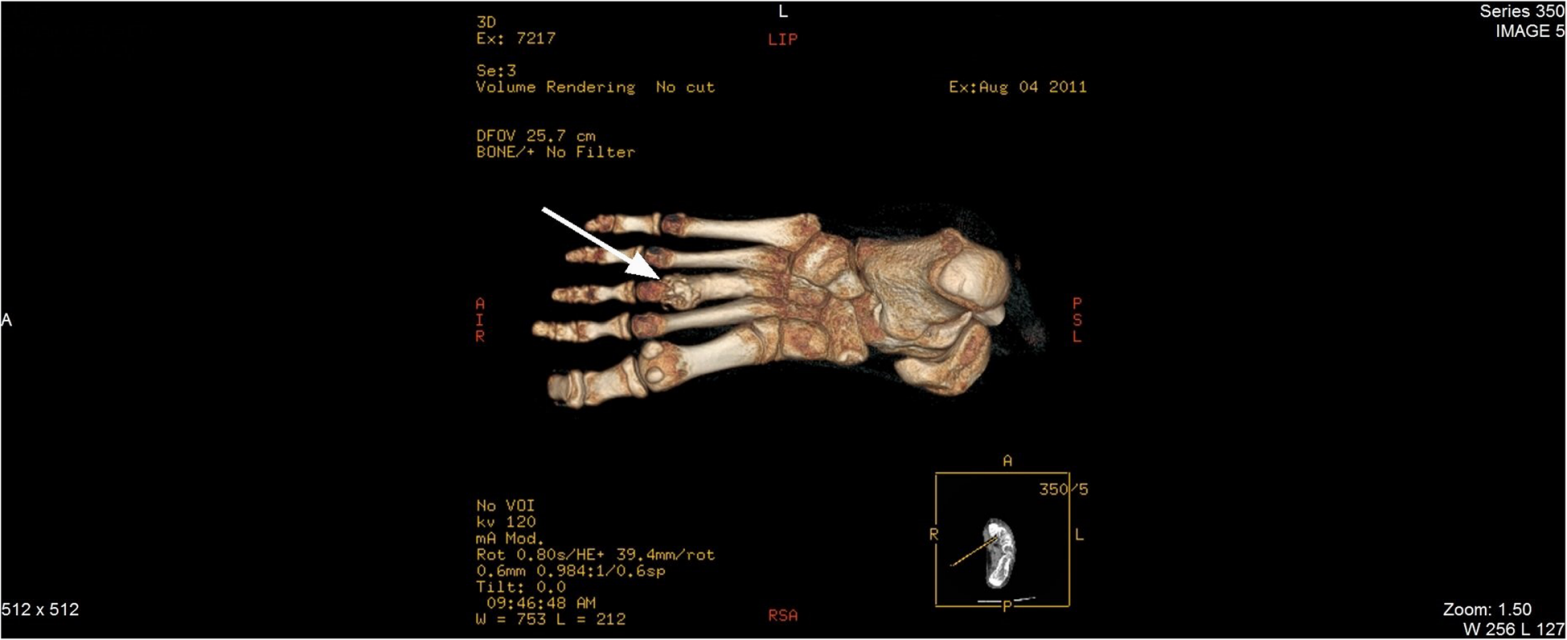
Three-dimentional reconstruction CT of the left foot. The destruction and sclerosis of third metatarsus bone diaphysis and distal metaphysis parts are indicated by white arrows

**Fig. 5:**
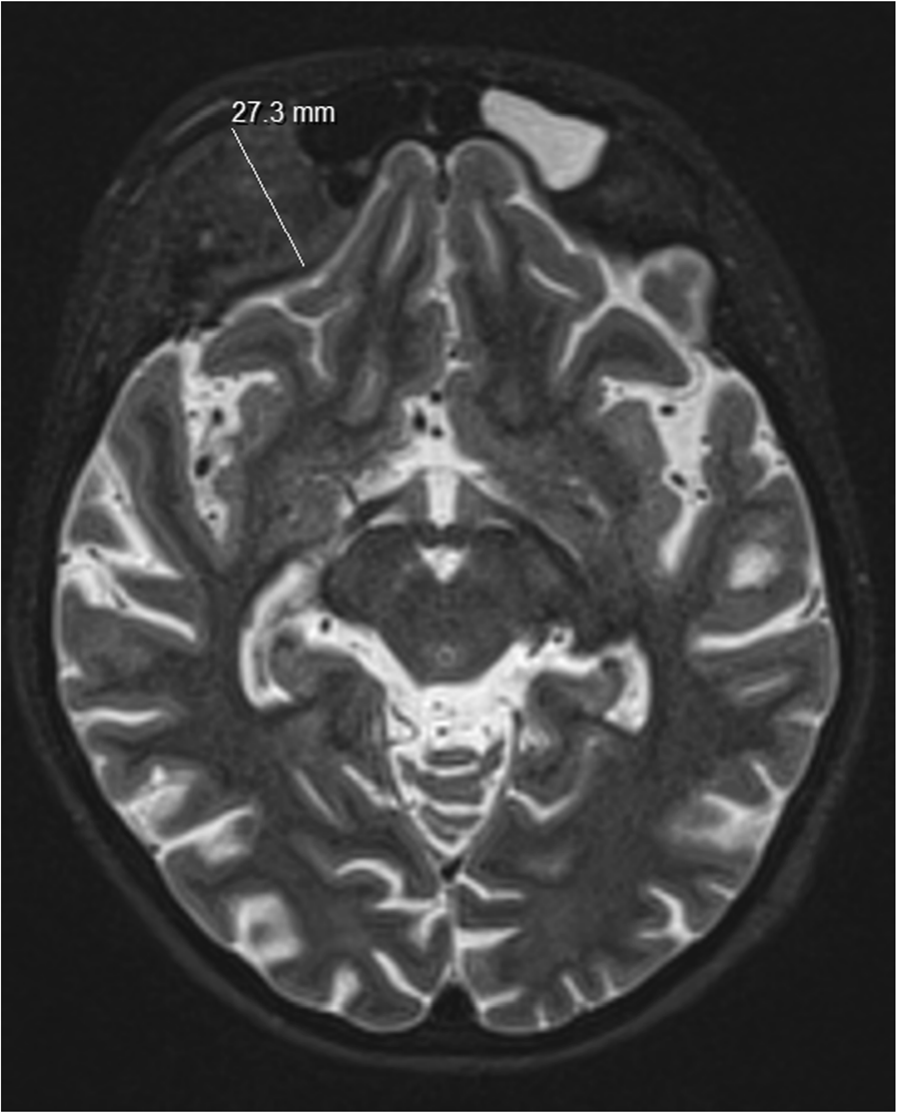
MRI of the patient‘s head. Measuring of hyperostosis of frontal bone on the right is shown by a white line

**Fig. 6:**
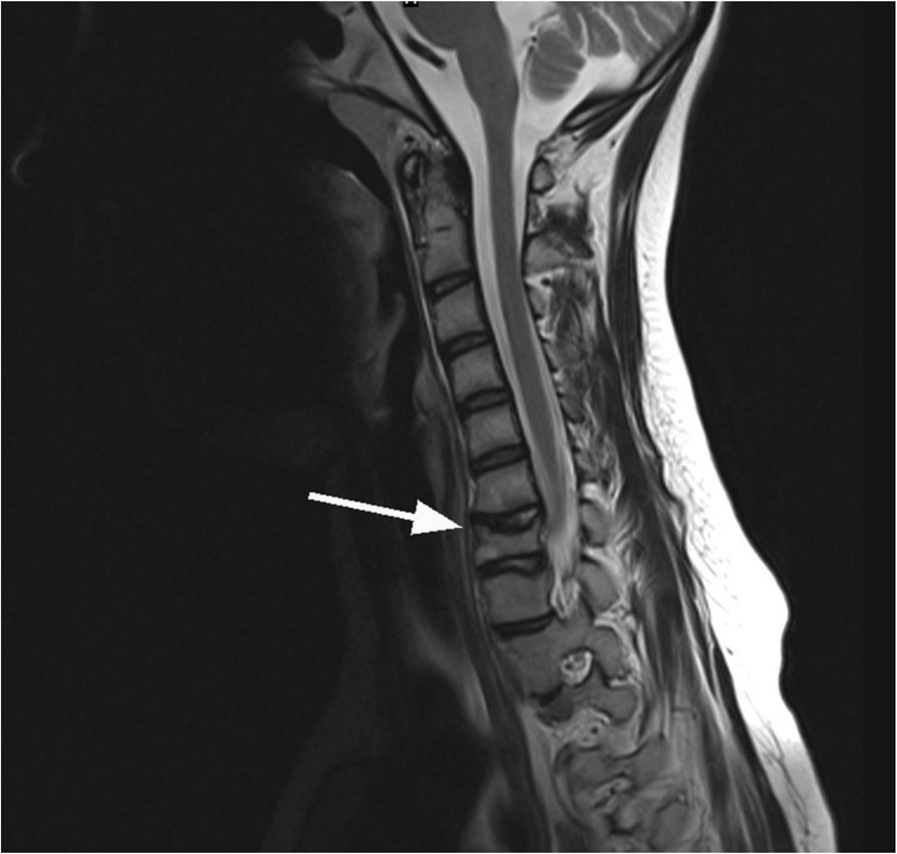
MRI of the patient’s neck. Destruction and lipoid degeneration in the seventh neck vertebrae is indicated by a white arrow

Since multiple bone lesions were detected, the fracture history was reviewed. At the age of 8 years our patient had not only a foot fracture, but also complained of pain in the left elbow and back. It appeared that she was diagnosed with CRMO at that time, based on the results of a biopsy from the fracture site as well as a CT of the left foot (Fig. 4) and a whole-body radionuclide scan. The radionuclide scan showed multiple sites of CRMO lesions, with three in the spine, one in the left hand, one in the left scapula, one in the left pelvic bone and two in the right leg. Surgery for the broken foot was performed and treatment with antibiotics and nonsteroidal anti-inflammatory drugs (NSAIDs) was prescribed for a month. When the patient’s pain decreased over time, analgesics were discontinued due to the impression of a self-limiting disease course. For the next 6 years she was free from complaints except for painless unilateral right periorbital oedema appearing on and off for the last 2–3 years and observed without medical supervision. Due to this oedema and slight exophthalmos of the right eye during current episode, additional head and spine magnetic resonance imaging (MRI) was performed, and a new CRMO lesion in a right periorbital region was diagnosed (Fig. 5) together with deformation and lipoid degeneration of the seventh neck vertebrae (Fig. 6).

Treatment was started for the vasculitis with cyclophosphamide and prednisolone according to the renal-limited vasculitis management protocol. No pain reliever was necessary. Severe metabolic disturbances and hyperparathyroidism were treated with alfacalcidol, calcium and magnesium supplements. Kidney and ureter stents were placed for 1 month due to hydronephrosis. Secondary GN-associated hypertension was treated with ACE (angiotensin converting enzyme) inhibitors. Anticoagulants were prescribed for deep vein thrombosis for 6 months until full recanalisation of the left femoral vein had occurred.

After 1.5 years of treatment, the child is free of complaints. Her auto-inflammatory, metabolic and endocrinological conditions are being monitored by a multidisciplinary team of physicians. All microelements and parathormone levels are within the normal range. There are no signs of hypertension and the patient’s kidney function has recovered. To date, there are no clinical or diagnostic signs of deep vein thrombosis. The patient is still on maintenance treatment with azathioprine according to the kidney vasculitis treatment protocol and receiving microelements supplementation.

## Discussion

Chronic recurrent multifocal osteomyelitis has been recognized as a disease entity for several decades. It is a multi-faceted disease. The diagnosis is based on clinical signs, radiological findings and bone biopsy results. There are several diagnostic criteria suggested for this condition [3, 20], the latest ones are by Roderick et al. [20]. According to Zhao et al., these guidelines should be followed in clinical practice and their application in typical cases can prevent or minimise the need for bone biopsy [21]. Still, there is no unified protocol to identify and characterise this disease [22]. Regarding our case, biopsy results were essential, as various bone lesions and deformities were found together with highly elevated inflammation markers. Furthermore, possible oncological conditions such as osteoblastoma, osteosarcoma and others had to be ruled out [23]. Bone biopsy revealed all stages of bone inflammation, with fibrosis detected together with the presence of plasma cells (representing adaptive immunity) [24] and neutrophils (responsible for innate immune activation) [24]. These findings, together with symptomatic C-ANCA-associated kidney vasculitis and deep vein thrombosis, further illustrated the immune system disturbances.

Currently, there are an increasing number of CRMO cases associated with other auto-inflammatory and autoimmune conditions. According to different data, we found two more cases of CRMO that eventually developed GPA [14, 15]. However, both cases demonstrated typical signs of respiratory tract involvement. Our case presented with isolated C-ANCA-associated kidney vasculitis without respiratory tract injury by otorhinolaryngological examination or lung CT scans. Our case fulfils three criteria out of six for GPA diagnosis proposed by EULAR/PRINTO/PRES [25]. But the question remains if granulomatous lesions in bones are an expression of a separate skeletal inflammatory disorder which eventually caused kidney vasculitis (as a complication of persistent chronic inflammation) or if this sporadic CRMO could be the musculoskeletal expression of GPA [26]. The recent studies of CRMO pathogenesis show a disturbed balance between pro- and anti-inflammatory cytokines such as interleukin 6 (IL-6), tumour necrosis factor alfa (TNFα) and interleukin 10 (IL-10) [24, 27]. Also, a reduced number of IL-10 producing cells in ANCA-associated vasculitis was shown in a study by Wilde et all*.* [28]. It may suggest a possible connection between these two conditions.

Moreover, it is reported that persistent inflammation and endothelial dysfunction associated with ANCA vasculitis increases the rate of thrombotic events and is linked to the active disease phase [29, 30]. Our patient‘s kidney biopsy results showed active vasculitis. Also, she had nephrotic proteinuria, which is known to increase the risk of thrombosis [31]. However, tests for protein C, protein S or antithrombin deficiencies, antiphospholipid antibodies and factor V Leiden mutation were negative in this case, indicating the possibility of other hypercoagulation mechanisms. Several hypotheses have also been proposed for activation of the coagulation cascade when well-known hypercoagulable risk factors remain undetected, including necrosis of endothelial cells and their circulation in the blood, wide-spread endothelial dysfunction, or thrombocyte activation [30].

Interestingly, together with CRMO bone lesions, there were a few cells positive for IgG and IgG4 identified in the bone biopsy, pointing to another rare pathology, IgG4-related disease. To date, there are only 25 paediatric case reports of IgG-related disease identified and they usually involve ocular and parenchymal organs with positive histological results for IgG4 cells [32]. Although the IgG4 levels in the blood were slightly elevated compared to the reference values (Table 4), no other symptoms or signs of organ injury were detected in our patient. Also, the kidney histological examination was negative for IgG cells. A few studies have shown that increased IgG4 levels in serum can be found in a wide spectrum of diseases, including vasculitis [33, 34]. This could be the reason in our case too.

Our patient had several severe metabolic deficiencies including hypocalcaemia, hypomagnesemia and hyperparathyroidism leading to osteoporosis. In relation to these findings, the possibility of other metabolic bone diseases (like Camurati-Engelman syndrome and benign hyperostosis-pachydermoperiostosis) was considered. However, the lack of particular skin lesions and MRI results strongly supported CRMO diagnosis not the other two diagnoses. Information about the relationship between CRMO and calcium metabolism is still lacking.

We also considered a few possible conditions that could have caused hyperparathyroidism in our case. First, we think that the hypocalcaemia may have developed due to the kidney damage and calcitriol production disturbances, which is well known in secondary hyperparathyroidism. This theory could be supported by the renal insufficiency and vitamin D3 hypovitaminosis that lasted for an unknown period. As our patient was free of complaints, her serum creatinine and vitamin D3 levels were not checked for several years. On the other hand, it is less likely that only renal insufficiency could have had influence on calcium metabolism as it was not severe on admission. Finally, as shown by Ata et al. [35], a high turnover rate of bone metabolism could be the most probable explanation for osteoporosis and hyperparathyroidism in this CRMO case. Closer follow-up of calcium metabolism might have provided an earlier hint of hyperparathyroidism and renal insufficiency due to the multiple bone lesions diagnosed after the first episode of CRMO 6 years ago.

Chronic recurrent multifocal osteomyelitis is a very versatile disease. Several studies have been done to identify the possible connection between immunological defects and gene mutations. Different data show that genetic metabolism errors are responsible for some of CRMO syndromes [7, 16], for example, the *PSTPIP1* gene is associated with pyogenic arthritis, pyoderma gangrenosum and acne (PAPA) syndrome [7] as well as defects in *LPIN2* result in Majeed syndrome [16]. A few research groups suggested a link between sporadic CRMO and gene errors along the 18q chromosome [36]. However, more studies are needed. There is no data showing a specific genetic defects in most of the sporadic cases, especially those associated with other conditions like GPA, dermatomyositis and other auto-immune diseases. Genetic testing for the *PSTPIP1* gene mutation was performed for our patient and was negative.

Despite various attempts to understand CRMO pathogenesis and favour diagnostic processes, no auto-antibody production has been observed to date, thus supporting an auto-inflammatory pathogenic mechanism of this condition [24, 37, 38]. We did not observe high auto-antibody titres in our case too. However, several different auto-antibodies were found. Anti-PCNA antibodies are known to occur in Sjogren’s syndrome [39], but no clinical signs of ocular or oral symptoms were present for this patient [40]. Also, we detected the presence of anti-CENP B antibodies, which are usually seen in systemic sclerosis [41]. Even so, our patient had no Raynaud’s phenomenon, skin lesions or other signs of systemic sclerosis. Moreover, an inflammatory processes and not antibody production was predominant throughout the course of the disease.

Recent studies of CRMO have led to a better understanding of pathogenic mechanisms and contributed the treatment improvements as following. NSAID monotherapy is thought to influence osteoclast activation by inhibiting prostaglandins, and thus impacting bone remodelling [24]. Moreover, glucocorticoids were shown to be important in stopping the general inflammatory response, thus having a positive effect in some cases [2]. Considering recent studies about bone remodelling in CRMO [35] and previous reports about increased osteoclast activity in bone histology specimens [42, 43], bone resorption inhibitors may be an effective treatment option. Moreover, bisphosphonates are strong osteoclast activity inhibitors and they may reduce bone turnover. Furthermore, they have shown to be effective in recurrent cases and in cases unresponsive to first-line medication [44, 45]. In very severe cases, biological therapy, for instance IL-1 or TNF-α inhibitors, have had a beneficial effect and limited progress of the disease [24, 46].

Our patient did not feel any pain and physical disability close to the CRMO lesion sites. Moreover, no vertebral fractures were detected to date and no additional treatment for CRMO was prescribed. Only treatment for vasculitis with a continuous low dose of prednisone and high doses of calcium and alfacalcidol supplements for the osteoporotic changes were given.

According to clinical guidelines, pain relief of the affected area is thought to be one of the most important criteria for clinical improvement. Despite no specific treatment, our patient had no complaints for 6 years after the initial diagnosis. Still, the progression of CRMO was obvious with a new lesion present in the MRI and painless bone deformities as a complication of the chronic course of the disease. Interestingly, this phenomenon has been described before [47] showing that active CRMO can be found in MRI, both, in periods without complaints and after various treatment options, even more than 10 years after the initial bone injury. Regardless of this data, it is still unknown if these clinically undetectable and painless lesions have clinical relevance to the disease progression and how long the treatment should be administered, i.e. to a complete radiological remission or improvement of clinical and laboratory signs [47]. Having this in mind and seeking to monitor possible severe complications (like vertebral fractures in the absence of complaints) the whole-body MRI with short tau inversion recovery (STIR) images is currently preferred as it was shown to be of superior sensitivity for evaluating CRMO lesion activity [48]. Well-timed MRI could show bone oedema prior to the appearance of erosions and/or sclerosis [48, 49]. In our case, comparative bone scintigraphy was done in parallel to the one done 6 years ago. One new CRMO lesion site was found in the right orbital area. No negative progression was observed in the previously diagnosed lesions. The MRI of the right orbital area detected sclerotic changes without bone oedema, indicating a continuous inflammatory processes that had likely developed within months or even years when the patient was supposedly symptoms-free and in a benign course of the disease. Taking into account other cases in which the patients eventually developed C-ANCA-associated vasculitis, we suggest that patients with multiple CRMO lesions should be monitored closely regarding chronic inflammation despite no signs or symptoms of disease activity.

Recently, research on new biomarkers for CRMO diagnosis and monitoring CRMO progression has been done by Hofmann et al. It showed that some changes in interleukin levels (IL-6, IL-12, eotaxin and others) were significantly altered during the CRMO disease course, meaning they could potentially be used as CRMO activity markers [37]. In our opinion, these biomarkers would be especially useful in unusual cases with atypical clinical courses, when some monitoring tools, for instance the PedsCNO score [50], are difficult to apply objectively.

## Conclusions

This case report presents a rare case of a complex immunodysregulatory disorder with both auto-inflammatory and autoimmune elements. We hypothesize that the long lasting active inflammation of CRMO may induce an autoimmune response, resulting in a concomitant disease such as the C-ANCA-associated vasculitis in our patient. Any potential specific pathogenic relationships between these two rare diseases may need to be further studied. Furthermore, there is still a lack of specific biomarkers for CRMO and more studies are necessary to identify CRMO’s characteristic patterns and how to best monitor disease progression.

## Abbreviations

ACF: Angiotenzine converting factor
ANA: Antinuclear antibodies
ANCA: Anti-neutrophil cytoplasmic antibodies
anti-CENP B: Antibodies against centromere protein B
anti-PCNA: Antibodies against proliferating cell nuclear antigens
C-ANCA: Cytoplasmic anti-neutrophil cytoplasmic antibodies
CRMO: Chronic recurrent multifocal osteomyelitis
CRP: C reactive protein
CT: Computed tomography
ESR: Erythrocyte sedimentation rate
EULAR: European League Against Rheumatism
GN: Glomerulonephritis
GPA: Granulomatosis with polyangiitis
IgG: Immunoglobulin G
IgG4: Immunoglobulin G4
IL-10: Interleukin 10
IL-12: Interleukin 12
IL-6: Interleukin 6
MRI: Magnetic resonance investigation
NSAIDs: Nonsteroidal anti-inflammatory drugs
PAPA: Pyogenic arthritis, pyoderma gangrenosum and acne syndrome
PedsCNO score: Paediatric chronic nonbacterial osteomyelitis score
PRES: Paediatric Rheumatology European Society
PRINTO: Paediatric Rheumatology INternational Trials Organization
STIR: Short tau inversion recovery
TNFα: Tumor necrosis factor alfa
